# Acupuncture for Refractory Epilepsy: Role of Thalamus

**DOI:** 10.1155/2014/950631

**Published:** 2014-12-07

**Authors:** Shuping Chen, Shubin Wang, Peijing Rong, Junling Liu, Hongqi Zhang, Jianliang Zhang

**Affiliations:** ^1^Institute of Acupuncture and Moxibustion, China Academy of Chinese Medical Sciences, Beijing 100700, China; ^2^China General Meitan Hospital, Beijing 100028, China; ^3^School of Chinese Medicine, Hong Kong Baptist University, Hong Kong

## Abstract

Neurostimulation procedures like vagus nerve stimulation (VNS) and deep brain stimulation have been used to treat refractory epilepsy and other neurological disorders. While holding promise, they are invasive interventions with serious complications and adverse effects. Moreover, their efficacies are modest with less seizure free. Acupuncture is a simple, safe, and effective traditional healing modality for a wide range of diseases including pain and epilepsy. Thalamus takes critical role in sensory transmission and is highly involved in epilepsy genesis particularly the absence epilepsy. Considering thalamus serves as a convergent structure for both acupuncture and VNS and the thalamic neuronal activities can be modulated by acupuncture, we propose that acupuncture could be a promising therapy or at least a screening tool to select suitable candidates for those invasive modalities in the management of refractory epilepsy.

## 1. Introduction

Epilepsy is one of the most common primary disorders of the brain leading to plenty of daily neuropsychiatric disabilities. Though most epilepsies can be well controlled by the conventional antiepileptic drugs (AEDs), there are however adverse effects and long-term impacts of the medications. Furthermore, about one-third of patients are resistant to AEDs albeit some could benefit from the surgical interventions [1, 2].

Neuromodulation is becoming one of the most vigorous fields in neurosurgery, as evidenced by the growing efforts invoking electrical stimulation to treat refractory epilepsy, chronic pain, Parkinson's disease, and so forth. Generally, the aberrant brain activities can be restored via electrical stimulation at either the peripheral nerve(s) or the very brain itself. Neurostimulation approaches such as vagus nerve stimulation (VNS), deep brain stimulation (DBS), trigeminal nerve stimulation (TNS), and so forth are reported to provide some palliation for refractory epilepsies, but, with different risk profiles, relatively low clinical efficiency (~30%), and less likeliness of seizure freedom. Moreover and unfortunately, many patients remain totally unresponsive to such interventions [3, 4].

## 2. Acupuncture for Epilepsy 

In traditional Chinese medicine (TCM), epilepsy was first described in The Yellow Emperor's Classic of Internal Medicine (Huang Di Nei Jing), an ancient Chinese medical book which was compiled around 770-221 B.C [5]. Its etiology, from the point of view of TCM, is supposed to chiefly result from the* qi* disturbance and thereby evolved to be the excess of* yang* due to deficiency of* yin* (*qi*,* yin*, and* yang* are abstract concepts in the antique Chinese philosophy to describe the energy or essence vital to homeostasis and proper function and its dynamic change inside the body). Since TCM considers that Du Meridian or Governor Vessel (GV), which is located in the posterior midline arising from the perineum to the head, is to govern the whole* yang* of the body, therefore, the most often used antiepileptic acupoints are selected from this Meridian [6].

A large number of clinical studies have demonstrated that acupuncture produces favorable effects on varied types of epilepsy such as absence seizure, febrile convulsion, generalized clonic-tonic seizure, and even status epilepticus. The overall therapeutic benefits include the improvement of electroencephalogram (e.g., the reduction of spike wave, desynchronization, etc.) and the epileptic symptoms (decrease of the seizure frequency, shortness of attack episodes, etc.) [7–9], alleviation of the severity of status epilepticus [10], functional recovery, and life quality improvement [11, 12]. Two recent studies showed that transcutaneous auricular vagus nerve stimulation (ta-VNS), a procedure similar to electroacupuncture (EA) stimulation at ear acupoints, is effective in reducing seizure frequency and improving the quality of life in pediatric as well as adult patients with refractory epilepsy, and the efficacy increases over the treatment time. In one study, among 47 epilepsy patients completed the 24-week treatment, six patients (12%) were seizure free, and 12 (24%) had a reduction in seizure frequency after 8-week session of treatment. At the timepoint of the week 16 of the continuous treatment, six patients (12%) were seizure free and 17 patients (34%) had a reduction in seizure frequency. After 24 weeks of treatment, eight patients (16%) were seizure free and 19 (38%) had reduced seizure frequency [13]. In the other study, we further verified the efficacy of ta-VNS for patients with refractory epilepsy. Among 98 patients recruited, after 8 weeks' treatment, 41.0% patients experienced reduction in seizure frequency that reached I, II, and III levels according to the standards of the Modified Engel Scale compared with the baselines, and the percentage of average seizure frequency was reduced by 42.6%. After 24 weeks of treatment, patients had 47.7% reduction of seizure frequency. Additionally, there were significant improvements in electroencephalograph (EEG) and the quality of daily life of the patients after treatment [14]. Compared to the conventional AEDs, the surgical interventions, and the neurostimulation procedures, acupuncture therapy is simple, safe, and less invasive [9, 15].

Animal studies have demonstrated the antiepileptic effects by acupuncture in varied animal models. It has been shown that EA remarkably depresses the cortical and amygdaloid epileptiform discharges and improves the epileptic electroencephalography [16–18] and attenuates the seizure behaviors in chemically induced experimental seizures [19]. Recent finding suggests that the EA-induced effects are acupoints and parameters dependent and usually appear 1 to 1.5 hours after the termination of EA stimulation [20]. Supportive evidence from dogs with idiopathic epilepsy which are nonresponsive to high levels of AEDs revealed that acupuncture leads to a change in seizure patterns and a reduction of the seizures [21]. Our own observation showed that EA produces ~27% reduction of pentylenetetrazol (PTZ) induced epileptiform neuronal activities of ventroposterior lateral thalamus of rat [22].

## 3. Neural Substrates for Acupuncture and VNS

### 3.1. Afferent Pathway of Vagus Nerve

**Figure 1:**
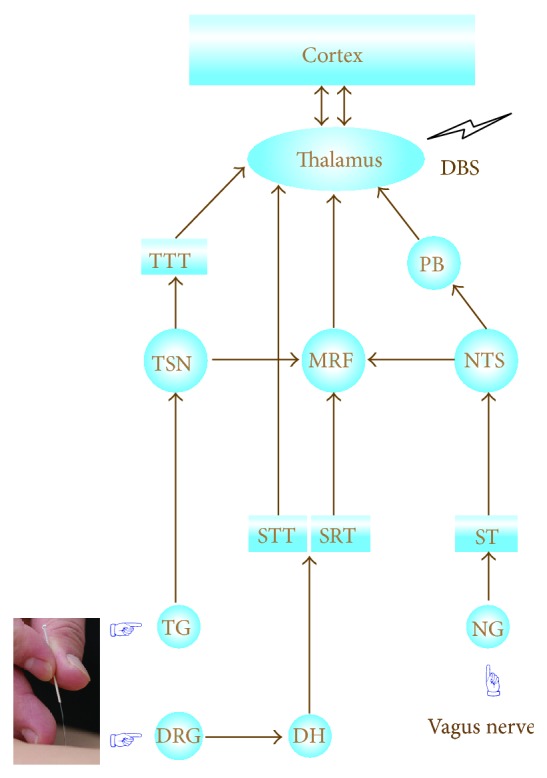
Schematic of neural substrates for VNS and acupuncture. (1) The vagus nerve carries information to the nodose ganglion (NG) and then relay mainly to bilateral nucleus tractus solitarius (NTS) via solitary tract (ST); thereby, the vagal afferents project densely to the parabrachial nucleus (PB) in the pons and other regions of the brainstem like medullary reticular formation (MRF) and numerous forebrain sites including limbic and olfactory cortices, hypothalamus (not specified), and the thalamus. (2) Acupuncture signals are generated from activation of skin receptors at acupoints and nearby tissues and then conveyed by *A*_*β*_, *A*_*δ*_, and *C* fibers to the dorsal horn (DH) of the spinal cord or trigeminal spinal nucleus (TSN) of the medulla. Then, they project to the thalamus via trigeminothalamic tract (TTT), spinothalamic tract (STT), and spinoreticulothalamic tract (SRT).

The vagus nerve is the most important parasympathetic nerve containing mixed afferent and efferent fibers in ratio of ~4 : 1. Its afferent direct and secondary connections are well established and are the most likely route of VNS brain effects. Briefly, the afferent sensory fibers carry information mostly from the head, neck, and abdominal organs to the nodose ganglion and then relay mainly to bilateral nucleus tractus solitarius (NTS), medullary reticular formation (MRF), the dorsal motor nucleus of the vagus, area postrema, and the nucleus cuneatus. From NTS, the vagal afferents project densely to the parabrachial nucleus (PB) in the pons, the dorsal raphe and numerous forebrain sites including infralimbic and olfactory cortices, amygdala, hypothalamus, and hippocampus as well as the thalamus (via PB) (Figure 1) [23–25]. In addition to those of visceral origin, vagus nerve has some somatic afferent fibers that innervate the inner (canal) portion of the outer ear, the so-called Arnold's nerve. Currently, a device is available which targets this auricular branch to achieve the antiseizure effects [26].

### 3.2. Conduction Route of Acupuncture Signals

As a physical sensory stimulus, manipulation of acupuncture needle certainly activates the somatic sensory receptors in particular the mechanoreceptors, which are sensitive to physical distortion like bending and stretching. The amplitude and magnitude of the acupuncture-induced signals vary with the manipulation modes, intensities, and individual differences in acupuncture sensitization of the subjects. Generally, signals from activation of skin receptors at acupoints and nearby tissues are conveyed by *A*_*β*_, *A*_*δ*_, and *C* fibers to the dorsal horn of the spinal cord or trigeminal spinal nucleus of the medulla. For signals originating from the body and extremities, specifically, they ascend via the spinothalamic tract directly or spinoreticulothalamic tract indirectly and project to the thalamus [27, 28], whereas for those from the head and face which are mostly innervated by the trigeminal nerve, acupuncture signals are brought to the trigeminal nucleus and then to the thalamus via trigeminothalamic tract. Eventually, information is relayed from thalamus to the cortex (Figure 1).

### 3.3. Cerebral Substrates Common to Acupuncture and VNS

It has been well established that both acupuncture stimulation and VNS can induce robust change of brain activities in a broad area of cerebral structures. Functional magnetic resonance imaging (fMRI) evidence showed that acupuncture activates the sensorimotor cortical network, including the insula, thalamus, anterior cingulate cortex, and primary and secondary somatosensory cortices, and deactivates the limbic-paralimbic neocortical network such as the medial prefrontal cortex, caudate, amygdala, posterior cingulate cortex, and parahippocampus [29]. Similarly, fMRI also identified some transcutaneous VNS-induced immediate and long-term BOLD (blood oxygen level dependent) changes in cerebral structures like thalamus, limbic system, and so forth that are overlapped with that of acupuncture [30]. Apart from the cortex and the diencephalon, there are two major convergent sites of afferent signals from acupuncture and VNS in the brainstem. One is MRF, which consists of more than 100 small neural networks, including the raphe nuclei, the magnocellular nuclei, and the parvocellular nuclei, and is essential in governing varied functions of the body such as pain regulation and cardiovascular control (Figure 1). The other is PB, which receives inputs, in addition to the heavy projections of NTS, from the trigeminal spinal nucleus (TSN) as well as the spinal dorsal horn, and is also a critical locus shared by acupuncture and VNS for their upward transmission of information (not specified in Figure 1). These convergent neural substrates support the speculation that acupuncture can potentially achieve comparable antiseizure effects of VNS and further, a synergistic action might arise from the combined use with both.

## 4. Thalamus Is the Convergent Pivot for Epileptogenesis, Acupuncture, and Neurostimulation

### 4.1. Role of Thalamus in Epilepsy Genesis

The thalamus is regarded as a gateway and switchboard of the sensory information transmission except the sole exception of olfaction and a powerful “pacemaker” for the massive input to the cerebral cortex. Likewise, the cortex has intimate connections and sends reciprocal descending projections in a complex and tightly coupled manner to the thalamus. The corticothalamocortical circuits mediate the generation of neural oscillations, and the abnormal hypersynchronized oscillations in the thalamocortical (TC) network, consisting of positive and negative feedback connections between the cortex and the thalamus, have been implicated as an underlying mechanism for the generation of the spike-wave discharges (SWDs), which is a characteristic of the absence epilepsy. It has been demonstrated that stimulation of the midline thalamic structures or microinjection of neuron analeptics to the ventrobasal thalamus elicited EEG responses in the cortex very similar to the SWDs of the generalized absence epilepsy [31, 32]. Recent evidence revealed that the phospholipase C beta4 (PLC*β*4) pathway tunes the firing mode of TC neurons via the simultaneous regulation of T- and L-type Ca(2+) currents as PLC*β*4-deficient TC neurons were readily shifted to the oscillatory burst firing mode after a slight hyperpolarization of membrane potential. TC-limited knockdown as well as whole-animal knockout of PLC*β*4 induced spontaneous SWDs with simultaneous behavioral arrests and increased the susceptibility to drug-induced SWDs, highlighting the role of thalamic PLC*β*4 in the genesis of absence seizures [33]. Additionally, the thalamus, for its diffuse connections with diverse regions of the cortex, may be partly responsible for the spread of seizures.

The massive TC synchronization is supposed to be driven by the recurrent oscillatory activities in the network between reticular thalamic nucleus (RTN), a thin layer of inhibitory *γ*-aminobutyric acid-containing neurons (GABAergic) adjacent to the thalamus, and TC relay nucleus [34–36]. The important element controlling the generation of activity in this system is the amplitude and duration of inhibitory postsynaptic potentials (IPSPs) in TC neurons, which depend on the pattern of activity generated in RTN cells [37, 38]. While the major projections of RTN neurons are onto relay neurons in dorsal thalamus, recurrent collaterals also provide intranuclear inhibition, which has been presumed to regulate RTN inhibitory output during thalamic oscillations and prevent the hypersynchrony of generalized absence epilepsy [38–40]. In addition, RTN receives the vagal information via the medulla counterparts to exert strong influence on the synchronization of TC projections [41]. Study on brain slices revealed that the GABAA receptor *β*3 subunit plays a vital role in controlling TC synchronization as in *β*3 knockout mice, the GABAA-mediated inhibition was nearly abolished in RTN, and the oscillatory synchrony was dramatically intensified [34].

### 4.2. Thalamus and Acupuncture Effects

#### 4.2.1. Electrophysiological, Neurochemical, and Molecular Evidence

The thalamus is also a key cerebral structure to mediate the acupuncture effects. By means of the extracellular recording technique, it was found that, in normal condition, electrical stimulation of the “Zusanli” acupoint (ST36, number 36 of the stomach meridian which is located below head of right fibula under knee joint and lateral to the anterior tubercle of the tibia) could activate the thalamic nucleus submedius neuronal activities though higher intensity is needed than that of the peroneal nerve stimulation, and the neuronal response properties were similar to both of the stimuli [42]. However, EA stimulation could significantly inhibit the nociceptive neuronal responses of parafascicular nucleus and nucleus ventralis posterolateralis (VPL) thalamus in rats, and this inhibitory effect was reduced or abolished by lesion of the cortical somatosensory area I (SI) or topical application of lidocaine to the cortical somatosensory area II (SII), indicating that SI and SII were involved in the descending modulation of acupuncture effect [43–46]. In addition, it was found that acupuncture could promote the release of cholecystokinin-like immunoreactivity and reduce the muscarinic receptors total binding capacities in the thalamus of rats [45, 47]. Our previous study showed that EA stimulation of “Dazhui” acupoint (GV14, number 14 of Governor Vessel which is located in the depression between the 7th cervical and 1st thoracic vertebra along the posterior midline) could predominantly inhibit the PTZ-induced epileptiform activities in the ventrobasal thalamic neurons. A typical extracellular recording demonstrated that EA stimulation (frequency: 30 Hz, current intensity: 1 mA, pulse width: 450 *μ*s, and duration: 5 min) at GV14 can almost abolish the PTZ-induced thalamic neuronal epileptiform activities and the neuron began to recover firing at the approximate timepoint of 750 s after the EA termination [22]. The latest study by means of gene microarray analysis revealed that acupuncture stimulation of GB34 (number 34 of gallbladder meridian, located in the depression anterior and inferior to the head of the fibula of the lateral side of the leg) and LR3 (number 3 of liver meridian, located in the depression of the posterior end of the 1st interosseous metatarsal space of the foot) could modulate the gene expression of the thalamus in model mice with parkinsonism [48].

#### 4.2.2. Neuroimaging Evidence

Neuroimaging directly demonstrated the vigorous involvement of thalamus in acupuncture or even laser acupuncture effects in terms of different acupoints and modes of stimulation [49, 50]. In rhesus monkeys, positron-emission tomography (PET) study showed that thermal nociception induced increase of regional cerebral blood flow (rCBF) of thalamus and EA stimulation at ST36 and LI4 (number 4 acupoint of large intestine meridian, which is located between the 1st and 2nd metacarpal bones of the dorsum of the hand) could suppress this activation [51]. Chen et al. reported that electrical stimulation near the median nerve could significantly attenuate the increased rCBF induced by amphetamine in the thalamus of rats [52]. They further extended the work to compare EA efficacy at different frequencies and locations and found that high frequency EA led to greater effects than low frequency EA did, and stimulation at forelimb acupoint (e.g., LI4) brought about greater effect than that of hindlimb (e.g., ST36) [53]. On the contrary, by simultaneously measuring the blood oxygenation level dependent (BOLD) and CBF, Zhang et al. found that high frequency EA stimulation of LI4 acupoint at innocuous intensity can induce activation of thalamus in human subjects [54]. In another study, manual acupuncture (MA) stimulation of BL60 (number 60 acupoint of bladder meridian located in the center of the depression between the prominence of the lateral malleolus of the fibula and the calcaneal tendon behind the ankle joint) can also elicit activating response in the thalamus [55].

#### 4.2.3. Thalamus Involvement in Diseases Treatment by Acupuncture

Particularly, the thalamus is implicated to be involved in acupuncture treatment for some pathological conditions. In patients with irritable bowel syndrome, it was demonstrated the enhanced thalamic activation during and immediately after EA, suggesting that acupuncture achieves its therapeutic efficacy via the ascending pathway through the thalamus [56]. Interestingly, for patients with functional dyspepsia, acupuncture induced deactivating response of the thalamus [57]. Notably, there were reports showing that acupuncture produced remarkable pain reduction and well-being improvement for patients with postapoplectic thalamic spontaneous pain [58, 59]. Overall, the thalamus is highly involved in acupuncture induced effects.

#### 4.2.4. Acupoints Prescriptions for Varied Epilepsies

As mentioned above, acupuncture produces broader therapeutic benefits on different types of epilepsy. Some acupuncture practitioners have summarized the acupoints prescriptions which are particularly effective in coping with certain specific epilepsies [11]. For instance, for grand mal, petit mal, and mixed epilepsy, acupoints including “Baihui” (GV20), “Yaoqi” (EX B9), “Neiguan” (PC6), “Jiuwei” (CV 15), and “Fenglong” (ST40) are usually selected for stimulation [60]. For general tonic-clonic epilepsy, however, in addition to GV20, CV15, EX B9, and ST40, other acupoints such as “Chengling” (BL18), “Yanglingquan” (GB34), and “Xinshu” (BL15) are often used [61]. For status epilepticus, a life-threatening condition with continuous and unremitting seizure, strong manual acupuncture stimulation is preferably applied at more acupoints of multiple meridians, individually or in combination. That is, except the abovementioned GV20, PC6, and ST40, acupoints such as “Renzhong” (GV26),“Hegu” (LI4), “Taichong” (LR3), “Yongquan” (KI1), “Jianshi” (PC5), “Shenmen” (HT7), “Guanyuan” (CV4), “Yintang” (EXHN3), “Fengchi” (GB20), and so forth are selected to stimulate prompt termination of the seizure attack [62]. Accordingly, for other types of epilepsy such as tonic-clonic epilepsy, Jacksonian epilepsy, and mixed epilepsy, there are corresponding specific acupoints and/or combinations for their treatments.

### 4.3. Thalamus Involvement in Neurostimulation Action

Neurostimulation procedures achieve their antiseizure effects directly or indirectly via thalamus. VNS is supposed to result in the antiseizure effects partly through inhibition of the thalamic relay centers as thalamus receives a large number of vagal projections [24, 63]. DBS (e.g., the anterior nucleus of the thalamus) has been proved to reduce seizures as such manipulation drives the cortical inhibitory circuits thereby leading to the increased short-interval intracortical inhibition and therefore produces beneficial effects on refractory epilepsy [64–66]. In addition, high frequency stimulation of cerebellum for refractory epilepsy is believed to activate the Purkinje cells which have an inhibitory effect on efferent projections to the thalamus [65, 67].

Brain imaging evidence obtained from patients with refractory epilepsy indicates the crucial role of the thalamus in the mechanism of action of VNS. Studies with the help of single-photon emission computed tomography (SPECT) revealed the inhibitory effects of VNS on thalamus. Ring et al. and Barnes et al. showed that the rCBF in thalamus was reduced in either chronic (with VNS for at least six months) or acute (no exposure to VNS prior to SPECT) conditions [68, 69]. This result was corroborated by the work of Vonck and colleagues who examined the effects of different stages of VNS implantation on thalamus and found that acute VNS leads to decreased rCBF in the left thalamus, bilateral parahippocampal gyrus, and right hippocampus, and chronic VNS caused a reduction of rCBF only in the left thalamus. However, when chronic activation was compared to chronic baseline, there was increase of rCBF in the left thalamus with no changes in other brain regions. This study highlights the complex time course of VNS-induced alteration of thalamic activity and its overall decrease over time, which may underlie the observation that VNS becomes more efficacious with time [70–73].

Contrary to SPECT, PET showed rCBF increase in the ipsilateral anterior thalamus in patients with VNS for epilepsy [74]. Another study by Ko et al., however, demonstrated that the increased rCBF occurred in the right thalamus, and the patient with the greatest clinic improvement by VNS had the highest increase of rCBF in the right thalamus [75]. Further study by Henry et al. demonstrated that both acute (20 hours after VNS ON) and long-term VNS (20 weeks of VNS) led to increased rCBF in the bilateral thalami in patients with clinical improvement [76, 77]. Consistent with PET evidence, fMRI studies revealed the robust activation in the bilateral thalami by VNS [78]. Notably, one study by Liu et al. reported that although VNS induced BOLD activation in a number of cerebral structures, only patients who showed clinical improvement in seizure occurrence had thalamic activation [79].

As a whole, abundant proofs suggest a major role of the thalamus underlying neurostimulation particularly VNS. With regard to the discrepancies of brain locations and activation directions across the PET, fMRI, and SPECT studies, it was hypothesized that this may be partly attributable to the different sampling time courses of these three imaging modalities. That is, SPECT has a 6–9 mm spatial resolution, 1-minute single acquisition time, and a 2-hour interacquisition interval, whereas PET has a 4-5 mm spatial resolution, ten second acquisition time, and 20–30-minute interacquisition interval. And unlike the above two which use the invasive gamma or positron emitting ligands, fMRI is based on the changes of concentration of oxyhemoglobin and has the least spatial resolution at 1-2 mm, a 2-3 second single acquisition time and 2-3 second interacquisition interval [80, 81].

## 5. Proposed Protocols for Evaluation of Antiseizure Efficacy by Acupuncture

Despite the substantial positive evidence, arguments remain regarding the use of acupuncture for epilepsy management [82]. It is very difficult, as a matter of fact, to design a randomized, double-blinded placebo-controlled study in patients with epilepsy. Hence, a systematic and well-controlled bench study is extremely important for validation of the efficacy of acupuncture before extrapolating its use in a clinical setting.

For plain evaluation of acupuncture efficacy, we suggest starting with one single acupoint to avoid potential interaction among multiple acupoints. Based on the documentary records and previous studies, GV14, the convergent acupoint of all six* yang* meridians of the body, is selected for stimulation with adequate frequency and modest intensity. Briefly, the study protocols are as follows. First, recruit participants and screen suitable candidates and make them consent, followed by eligibility evaluation with pretreatment, then randomize them to verum versus sham acupuncture groups for a session of six-month treatment, and finally, assess the acupuncture efficacy by calculating the seizure frequency and severity in number and evaluate the overall satisfaction and adverse events by semistructured interviews in the immediate end of treatment and twelve-month follow-up.

Several concerns, however, remain to be clarified in the subsequent study. First of all, the sample size, which once was a major limitation for the previous studies, should be appropriate. Second, it is suitable to focus on one certain type of epilepsy to avoid the heterogeneity of epilepsy patients. Third, the sham acupuncture, like the use of validated Streitberger sham acupuncture device and stimulation of acupoints unrelated to epilepsy treatment, should be deliberately performed in the control group [83].

## 6. Conclusion

The thalamus is a crucial structure in epilepsy generation and propagation as well as neurostimulation and acupuncture treatment. It is likely that afferent inputs by acupuncture, VNS, TNS, and so forth could evoke sufficient inhibition to modulate or interrupt, if optimal stimuli delivered, the pathological oscillation within the thalamus and the cortex attributable to seizure generation and propagation. We suppose that, on one hand, acupuncture, like neurostimulation procedures, can acutely depress or abolish the epileptiform thalamic neuronal activities, thereby, modulate the thalamocortical oscillation and, eventually, prevent seizure onset and propagation. The chronic effects by acupuncture, on the other hand, which probably result from repetitive stimulation, might be involved in modulation of synaptic plasticity, neurotransmitters metabolism, or even neural reconstruction via the intricate transcriptional and posttranscriptional regulations. Thus, it is rational to conclude that acupuncture could work as a promising therapy, or at least a screening tool, to select suitable candidates for these invasive modalities in the management of refractory epilepsy.
